# Supplementary material for: WordCommentsAnalyzer: A windows software tool for qualitative research
Source: F1000Res. 2018 Sep 4;7:536. Originally published 2018 May 3. [Version 2] doi: 10.12688/f1000research.14819.2 (PMC6137411; doi:10.12688/f1000research.14819.2)
Supplement: Supplementary file 3 [file f1000research-7-17614-s0002.tgz › b2a79496-4ada-47c4-9682-a99ce867836d.zip!/Eight Iranian Health Journals/The Journal of Toloo-e-Behdasht_36.docx]

© The Journal of Toloo-e-Behdasht. This work is licensed under CC BY 4.0 (https://creativecommons.org/licenses/by/4.0/).

Volume & Issue: دوره 10 - شماره 3 و 3-4

Title: آسیب شناسی منابع انسانی در ستاد دانشگاه علوم پزشکی کرمان با استفاده از مدل سه شاخگی – 1389

http://tbj.ssu.ac.ir/article-1-1796-fa.html

سابقه واهداف: با توجه به نقش مهمی که کارکنان ستادی در دانشگاه های علوم پزشکی دارند، دانشگاه ها باید همواره در جستجوی راه هایی برای توسعه نیروی انسانی ستادی خود باشند و گام اول در این مسیر آگاهی از وضعیت موجود نیروهای ستادی از طریق فرایند آسیب شناسی می باشد. پژوهش حاضر با هدف آسیب شناسی و برنامه ریزی توسعه منابع انسانی درحوزۀ ستادی معاونت های دانشگاه علوم پزشکی کرمان انجام شده است. روش بررسی:  پژوهش از نوع مقطعی توصیفی است.  159  نفر  از کارکنان  ستادی دانشگاه علوم پزشکی کرمان، در این پژوهش شرکت کردند.  اطلاعات لازم برای این پژوهش از طریق پرسشنامه به دست آمد. تحلیل داده هابا استفاده از آزمون های فریدمن، مجذور کای  وتی- تست به کمک نرم افزار spss انجام گردید. یافته ها : عوامل رفتاری( میانگین33/2) بیش ترین تاثیر وعوامل زمینه ای(میانگین 24/3) کم ترین تاثیر را درآسیب های منابع انسانی دانشگاه علوم پزشکی کرمان داشتند. ازبین زیرمجموعه های عوامل رفتاری، انگیزش ورضایت شغلی ( میانگین رتبه 27/2)بیشترین تاثیر رادرایجاد آسیب های منابع انسانی داشته است و مشتری گرایی( میانگین رتبه 77/1) در مقایسه با زیر مجموعه آسیب ها در هر سه شاخه کمترین تاثیر را در آسیب های منابع انسانی داشته است. نتیجه گیری: با شناخت آسیب های منابع انسانی در واحدهای ستادی دانشگاه علوم پزشکی کرمان، به نظر می رسد که  مشکلات فوق محدود به دانشگاه علوم پزشکی کرمان نبوده و نظر به ساختاری بودن این عوامل، معاونت سرمایه انسانی و معاونت برنامه ریزی ریاست جمهوری می تواند با بازنگری در نظام های پرداخت و سیستم ارزیابی عملکرد تحولی اساسی را در دانشگاه های علوم پزشکی با امید افزایش انگیزه کارکنان فراهم آورد.

Title: بررسی تعیین کننده های قصد بستگان مبتلا به سرطان کولورکتال به انجام غربالگری بر اساس تئوری رفتار برنامه ریزی شده

http://tbj.ssu.ac.ir/article-1-1795-fa.html

چکیده سابقه و اهداف:سرطان کولورکتال یکی از سرطان های شایع درجهان  می باشد که در کشورهای پیشرفته سومین سرطان شایع و دومین سرطان کشنده می باشد.احتمال ابتلا به این بیماری در بستگان درجه یک مبتلا به سرطان کولورکتال 2 الی 3 برابر بیشتر می باشد.برنامه ریزی در جهت پیشگیری در این گروه نیازمند بررسی نیازهای فعلی آنها و ارزیابی میزان آگاهی و قصد آنها نسبت به  پیشگیری می باشد. بنابراین، این بررسی با هدف تعیین قصد بستگان مبتلا به سرطان کولورکتال بر اساس تئوری رفتار برنامه ریزی شده در شهر یزد انجام شد. روش بررسی:در این مطالعه توصیفی تحلیلی تعداد99 نفر مرد وزن بالای 20 سال از اقوام درجه یک  بیماران سرطان کولورکتال بصورت سرشماری  وارد مطالعه شدند.ابزار جمع آوری داده ها پرسشنامه ای 45 آیتمی بر اساس  استفاده از تئوری رفتار برنامه ریزی شده  بود. داده ها با استفاده از آزمونهای همبستگی پیرسون ،آزمون Anova تعقیبی توکی ورگرسیون چندگانه و T-test مورد تجزیه وتحلیل قرار گرفت. یافته ها: حدود9/65%(59 نفر) از شرکت کننده های این مطالعه مرد و 4/40%(40 نفر) زن بودند که میانگین سنی آنها 39 سال بود.میانگین نمره آگاهی 3±57/10 از 19،میانگین نمره نگرش46/9±39/63 از 90، میانگین نمره قصد رفتار5/3±35/8 از 15، میانگین نمره هنجار انتزاعی14/3±13/9 از 15، میانگین نمره کنترل رفتار درک شده 97/1±31/ 5 از 10بود. سازه های آگاهی، نگرش، هنجار انتزاعی و کنترل رفتار درک شده تا 73 درصد قصد به انجام غربالگری را پیشگویی می کنند. نتیجه گیری: میزان آگاهی،نگرش،هنجار انتزاعی،کنترل رفتار درک شده و قصد در سطح پایین بود .با توجه به سطح پایین این سازه ها  لازم است با آموزش آنها ازطرق گوناگون براساس تئوری ها و الگوهای رفتاری  از جمله تئوری رفتار برنامه ریزی شده قصد بستگان را نسبت به انجام غربالگری  افزایش داد.    این مقاله برگرفته از پایان نامه دوره کارشناسی ارشد آموزش بهداشت دانشگاه علوم پزشکی شهیدصدوقی یزد می باشد.

Title: بررسی ارتباط افت فشار و ظرفیت حذف هیدروژن سولفاید با بار ورودی در بیوفیلتراسیون با بستر لجن فعال و مخلوط لجن فعال با سیلیکای شلتوک برنج

http://tbj.ssu.ac.ir/article-1-1797-fa.html

چکیده سابقه و اهداف: در سالهای اخیر محققین نشان دادند که افزایش افت فشار و پایین بودن ظرفیت حذف آلاینده ها از محدودیتهای سیستمهای بیوفیلتراسیون می باشند، بنابراین هدف اصلی این مطالعه بررسی ارتباط بین میزان افت فشار و ظرفیت حذف هیدروژن سولفاید (H2S) با میزان بار ورودی  در سیستم بیوفیلتراسیون با کاربرد دو بسترلجن فعال خشک و مخلوط لجن فعال با سیلیکای حاصل از شلتوک برنج بوده است. روش بررسی: دو فیلتر از جنس پلی وینیل کلراید به حجم هر فیلتر 1 لیتر طراحی و ساخته شد. لجن فعال خشک از تصفیه خانه فاضلاب شهری پوتراجای مالزی تهیه گردید. سیلیکای شلتوک برنج پس از اسید شویی و احتراق با حرارت 800 درجه سانتی گراد  بمدت 4 ساعت در کوره تهیه شد. سیستم با زمان ماندهای خالی( (EBRT مختلف از 30 تا90 ثانیه و بارهای ورودی H2S از 36/18 تا 54 gm-3h-1 مورد آزمایش قرار گرفت. یافته ها: این مطالعه نشان داده است که بر اساس آنالیز آماری همبستگی پیرسون بین افزایش بار ورودی H2S و میزان ظرفیت حذف در هر دو بستر رابطه مستقیم و قوی وجود دارد(01/0>p). همچنین تحت شرایط 54 gm-3h-1 بار ورودی H2S و EBRT 30 ثانیه، ماکزیمم ظرفیت حذف H2S در فیلتر با بستر لجن فعال و بستر مخلوط لجن و سیلیکای شلتوک به ترتیب 33/44 و53/52 gm-3h-1 بوده است. علاوه بر این بر اساس آنالیز آماری همبستگی پیرسون، در هر دو بستر بین افزایش بار ورودی H2S و افت فشار یک رابط مستقیم و قوی وجود دارد(0.01>p). بطوریکه تحت شرایط با بار ورودی مختلف از 36/18 تا 54 gm-3h-1 و زمان های مختلف کارکرد فیلتر تا 53 روز، میزان افت فشار در صافی با بستر لجن فعال و بستر مخلوط لجن فعال و سیلیکای برنج به ترتیب 0/8 و 0/3 mm H2O بوده است. نتیجه گیری: مطالعه حاضر نشان داده است که هر چند بین افزایش بار ورودی H2S و ظرفیت حذف و افت فشار رابطه مستقیم و مثبت وجود دارد اما بستر مخلوط لجن فعال با سیلیکای شلتوک برنج دارای ظرفیت حذف بیشتر و افت فشار کمتری بوده است.

Title: بررسی میزان برخی آلاینده های داخل منازل روستایی شهرستان عجبشیر استان آذربایجان شرقی

http://tbj.ssu.ac.ir/article-1-1798-fa.html

سابقه و اهداف : مخاطرات بهداشتی ناشی از تماس با آلاینده های هوا در محیط های بسته، به مراتب بیش از فضای آزاد می باشد. این امر بیانگر توجه به مبحث آلودگی هوا در داخل محیط‌های بسته و نقش تاثیرگذار آن بر سلامتی افراد می‌باشد. هدف از این مطالعه بررسی میزان آلاینده های داخل منازل روستایی شهرستان عجبشیر استان آذربایجان شرقی می باشد.  روش بررسی: این مطالعه ی توصیفی مقطعی جهت بررسی کیفیت هوای داخل منازل روستایی، آلایند ه ها و تأثیرات متقابل نوع سوخت مصرفی و وضعیت تهویه و آلودگی هوای داخلی بر یکدیگر، مقادیر آلاینده‌های دی‌اکسید نیتروژن (NO2)، منواکسید کربن (CO)، دی اکسید سولفور(SO2) و ذرات معلق هوا(PM10) در منازل روستاهای تپیک دره و خانیان در شهرستان عجبشیر طی مدت یکسال توسط دستگاههای مخصوص سنجش آلاینده ها مورد سنجش قرار گرفت. اطلاعات بدست آمده جمع آوری و با استفاده از نرم افزارSPSS وآزمونهای آماری و آنالیزواریانس در سطح احتمال 5% مورد بررسی قرار گرفت، نتیجه بصورت درصد فراوانی و میانگین همراه با میزان انحراف از استاندارد بیان گردید.  یافته ها: از تجزیه واریانس 112مورد مقادیرآلاینده های اندازه گیری شده در فصول مختلف برای روستای خانیان نتایج نشان می‌دهدکه غلظت آلاینده ها در  فصول مختلف سال تفاوت معنی‌دار(05/0&le P)  دارند. در جدول تجزیه‌ی واریانس روستای تپیک دره برای هر دو مورد ایستگاهها و فصول مختلف سال، تنها در مورد گاز  COاختلاف معنی‌دار مشاهده می گردد (01/0&le P). نتیجه گیری: نتایج این مطالعه نشان داد که به دلیل استفاده از سوختهای فسیلی و عدم رعایت بهداشت محیط و استفاده از تنورهای سنتی در داخل منازل روستایی، استفاده از سوخت‌های جامد،  استفاده از اجاق‌های روباز و فاقد دودکش در داخل منازل و وجود کارگاه قالیبافی در محل سکونت افراد خانوار میزان غلظت آلاینده‌های هوا در منازل روستایی بالا است و لازم است تدبیراتی در زمینه ی کاهش این آلودگی ها در این روستاها اندیشه شود.

Title: بررسی میزان غلظت بنزن در داخل کابین تاکسی های شهر یزد با استفاده از روش SPME

http://tbj.ssu.ac.ir/article-1-1800-fa.html

چکیده سابقه و اهداف: بنزن، به عنوان یکی از ترکیبات آلی فرار و سرطانزا، می تواند از انواع فوم ها و پلاستیک های موجود در کابین خودرو آزاد گردد. هدف از انجام این مطالعه تعیین  میزان غلظت بنزن موجود در کابین تاکسی های  شهر یزد می باشد. روش بررسی: مطالعه حاضر از نوع توصیفی بوده و در فصل بهار انجام شده است. که به شیوه طبقه ای-تصادفی تعداد30 تاکسی انتخاب و از هوای داخل کابین توسط پمپ نمونه برداری فردی و کیسه تدلار نمونه گیری گردید و نمونه ها  با روش میکرواستخراج از فاز جامد استخراج و با استفاده از دستگاه گاز کروماتوگرافی با دتکتور یونش شعله ای تجزیه و تعیین مقدار گردیدند. یافته ها: با توجه به نتایج، میانگین غلظت بنزن در کلیه تاکسی ها در وضعیت توقف بیشتر از وضعیت حرکت بود بطوری که میزان آن در وضعیت توقف 905 و در وضعیت حرکت 309 میکروگرم گرم بر متر مکعب بود و اختلاف میانگین ها نیز از لحاظ آماری معنی دار می باشد. همچنین بالاترین و پایین ترین غلظت بنزن داخل کابین در حالت توقف g/m3µ3200 و 122 و در وضعیت حرکت به ترتیبg/m3 µ1000 و g/m3µ57 بود. با توجه به یافته ها، میانگین غلظت بنزن در تاکسی های با عمر بیشتر از 6 ماه (g/m3µ82± 1138) بیشتر از تاکسی های با عمر کمتراز 6 ماه (g/m3µ 687± 671) می باشد ولی اختــلاف میانگین ها در دو وضـعیت توقف و حرکـت از لحـاظ آمـاری معـنی دار نمی باشد(05/0P>). نتیجه گیری:نتایج این مطالعه نشان می دهد که غلظت بنزن داخل کابین تاکسی های مورد مطالعه بالا بوده و از حد استاندارد نیز بالاتر می باشد. با توجه به این که در فصل تابستان درجه حرارت در اکثر نقاط کشور بالاتر  می باشد و با توجه به این که در اکثر نقاط پارکینگ مسقف پیش بینی نشده و یا تعداد آن ها کم می باشد لذا  خودروها عمدتاً در فضای باز و زیر آفتاب پارک می شوند و احتمال متصاعد شدن ترکیبات فرار و به ویژه بنزن افزایش می یابد.   این مقاله برگرفته از پایان نامه دوره کارشناسی ارشد بهداشت حرفه ای، دانشگاه علوم پزشکی شهیدصدوقی یزد می باشد.

Title: تحلیل روابط بین معنای معنوی با بهزیستی معنوی، شادی ذهنی، رضایت از زندگی، شکوفایی روانشناختی، امید به آینده و هدفمندی در زندگی دانشجویان

http://tbj.ssu.ac.ir/article-1-1799-fa.html

سابقه و اهداف: تعریف معنا در زندگی به اشکال متنوعی از انسجام و یکپارچگی در زندگی تا هدفمندی و برخورداری از جهت یا معناداری و اهمیت هستی شناسی از دیدگاه فرد تجربه کننده ارائه شده است.  هدف اصلی پژوهش حاضر تحلیل روابط بین معنای معنوی با بهزیستی معنوی، شادی ذهنی، رضایت از زندگی، شکوفایی روانشناختی، امید به آینده و هدفمندی در زندگی دانشجویان دانشگاه اصفهان و آزاد اسلامی خوراسگان بوده است. روش بررسی: جهت انجام این پژوهش از یک طرح پژوهشی همبستگی استفاده شد. جامعه آماری این پژوهش را کلیه دانشجویان دانشگاه اصفهان  و آزاد اسلامی خوراسگان در سال تحصیلی 1389-1388 تشکیل می دادند که به روش تصادفی طبقه ای و با استفاده از جدول تعیین حجم نمونه کوهن و مانیون و موریسون، تعداد 400 نفر  بعنوان حجم نمونه در نظر گرفته شدند. ابزارهای اصلی گردآوری اطلاعات در این پژوهش شامل مقیاس های معنای معنوی ماسکارو و همکاران، بهزیستی معنوی کارمودی و همکاران، شادی ذهنی لیبومیرسکی، رضایت از زندگی داینر، شکوفایی روانشناختی داینر و دیسواس- داینر، امید به آینده اسنایدر و همکاران و هدفمندی در زندگی اسچیر و همکاران بود.  پایایی مقیاس ها بر حسب ضریب آلفای کرونباخ به ترتیب 79/0، 87/0، 56/0، 60/0، 87/0، 82/0 و 74/0 و  روایی آنها  نیز به شکل صوری و محتوایی توسط متخصصین روانسنجی و موضوعی مورد تأیید قرار گرفت.  یافته ها: یافته ها نشان داد که بین معنای معنوی با دو زیر مقیاس بهزیستی معنوی(باور معنوی و آرامش معنوی)، شادی ذهنی، رضایت از زندگی، شکوفایی روانشناختی، امید به آینده و هدفمندی در زندگی رابطه مثبت و معنادار وجود دارد و بیشترین رابطه معنای معنوی با هدفمندی در زندگی دانشجویان می باشد. همچنین نتایج تحلیل رگرسیون چندگانه گام به گام گویای آن بود که در گام اول، هدفمندی در زندگی 38 درصد، در گام دوم با اضافه شدن باور معنوی 47 درصد، در گام سوم با اضافه شدن آرامش معنوی 50 درصد، در گام چهارم با ورود شکوفایی روانشناختی 52 درصد و در نهایت با افزودن امید به آینده 53 درصد از واریانس معنای معنوی تبیین می گردد. نتیجه گیری: به نظر می رسد که معنای معنوی به وسیله هدفمندی در زندگی، باور معنوی، آرامش معنوی، شکوفایی روانشناختی و امید به آینده تحت تأثیر قرار گیرد. به عبارت دیگر، زمانی که دانشجویان درک کنند که معناداری در زندگی شان مبتنی بر معنویت است، تلاش می کنند که هدفمند رفتار کنند.

Title: کاربرد فرایند فتوکاتالیستی TiO2/UV-C در حذف رنگ راکتیو قرمز 198 از فاضلاب سنتتیک نساجی

http://tbj.ssu.ac.ir/article-1-1801-fa.html

سابقه واهداف: رنگ بارزترین آلاینده فاضلاب های نساجی می باشد که به دلیل اثرات سوء بهداشتی و زیست محیطی مورد توجه جدی می باشند. رنگ ها موادی با ساختار پیچیده، معمولاً سمی و مقاوم به تجزیه بیولوژیک هستند که از طریق فاضلابهای صنعتی وارد محیط زیست می شوند. هدف این مطالعه تجزیه فتوشیمیایی(UV) و فوتوکاتالیستی(TiO2/UV-C ) رنگ راکتیو قرمز 198 بود. روش بررسی: این مطالعه بصورت تجربی و در مقیاس آزمایشگاهی در راکتور ناپیوسته انجام شد. فاضلاب سنتتیک از انحلال رنگ راکتیو قرمز 198 در آب شهر تهیه و تأثیر غلظت اولیه رنگ، جرم TiO2، pH  و زمان تماس در حذف رنگ بررسی شد. غلظت رنگ با استفاده از اسپکتروفتومتر در طول موج 518 نانومتر تعیین گردید. یافته ها:. فرایند TiO2/UV-C  نسبت به فرآیند UV-C تنها، موثرتر است. ثابت سرعت تجزیه فتوشیمیایی در pH اسیدی 0019/0 و طی فرایند تجزیه فتوکاتالیستی  0381/0 بر دقیقه می باشد. تجزیه نوری و فتوکاتالیستی رنگ مورد نظر از مدل سینتیک درجه اول پیروی می کند. افزایش جرم  TiO2از 4/0 به 1 گرم منجر به افزایش راندمان از 8/97 به 100 در صد می گردد. افزایش غلظت رنگ از 100 به 250 میلی گرم در لیتر و pH  از 4 به 10 منجر به کاهش راندمان به ترتیب  از 100 به 66/97 و از 100 به 6/94 می گردد. بهترین راندمان در pH معادل 4 حاصل می گردد. نتیجه گیری: بیشترین کارآیی حذف رنگ راکتیو قرمز 198 با فرایندهای اکسیداسیون فتوشیمیایی و فتوکاتالیستی تحت شرایط اسیدی حاصل می شود که از این میان فرآیند فتوکاتالیستی موثر تر می باشد.

Title: بررسی روند تغییرات کیفی و کمی منابع آب زیرزمینی دشت یزد- اردکان در دهه 88-1379

http://tbj.ssu.ac.ir/article-1-1803-fa.html

چکیده سابقه و اهداف: امروزه با افزایش جمعیت و افزایش نیاز آب در بخش­های مختلف کشاورزی، صنعتی، شرب و بهداشت، فشار زیادی به منابع آب­ زیرزمینی وارد شده است. این مسأله در کنار وقوع خشکسالی­های شدید و طولانی مدت شرایطی را برای کشور، خصوصاً برای مناطق خشک و فراخشک پدید آورده است که آن را با چالش­های جدی در زمینه کمیت و کیفیت آب مواجه کرده است. در این تحقیق، روند تغییرات کیفی و کمی منابع آب زیرزمینی و روند خشکسالی در دشت یزد- اردکان بعنوان یکی از مهمترین مراکز جمعیتی ایران مرکزی، مورد بررسی قرار می­گیرد. روش بررسی: به منظور بررسی تغییرات کیفی منابع آب زیرزمینی از داده­های کیفی دشت یزد اردکان در بازه زمانی 1379 تا 1388 مربوط به منابع انتخابی شرکت سهامی آب منطقه ای یزد استفاده شد و با نرم افزارهای آماری (Minitab) و کیفی آب(Aquachem)، این داده ها ارزیابی و روند تغییرات زمانی بررسی گردید. با استفاده از داده­های مکانی و توصیفی و بکارگیری  سیستم اطلاعات جغرافیایی­،  لایه­های اطلاعات مکانی برای همه پارامترهای کیفی آب زیر­زمینی دشت تهیه و برروی هم منطبق گردید و مورد ارزیابی قرار گرفت. همچنین به منظور برآورد شدت و مدت خشکسالی­ اقلیمی از شاخص استاندارد بارش (SPI) و جهت بررسی روند افت سطح آب زیرزمینی از هیدروگراف بلند مدت چاه های پیزومتری موجود در دشت یزد- اردکان استفاده گردید. یافته ­ها: نتایج بررسی تغییرات سطح آب زیرزمینی در 4 دهه اخیر حاکی از روند نزولی آن بوده و متوسط افت سطح ایستابی، حدود 5/0متر در سال می باشد. هم­چنین نتایج حاصل از تغییرات کیفیت آب در دهه اخیر نشان داد که با افزایش تکرار خشکسالی و افت شدید سفره آب زیرزمینی ، کیفیت آب زیر زمینی، خصوصاً در بازه زمانی 1385 تا 1389 دارای روند نزولی بوده است. نتایج نشان داد که در طی50 سال اخیر، تکرار وقوع خشکسالی اقلیمی در دهه اخیر نسبت به چهار دهه گذشته، 4 برابر شده است. نتیجه­ گیری: با توجه به نتایج می­توان گفت که تنها راه اساسی و اصولی جهت جلوگیری از عواقب خطرناک افت سطح ایستابی و کاهش کمیت و کیفیت منابع آب زیرزمینی، مصرف درست و قانونمند آب و جلوگیری از برداشت بی‌رویه آب­های زیرزمینی می­باشد.

Title: بررسی تاثیر رویکرد گروه همسالان بر آگاهی و عملکرد دختران نوجوان پیرامون سلامت روان

http://tbj.ssu.ac.ir/article-1-1802-fa.html

سابقه واهداف: بلوغ از مهم‌ترین دوره‌های زندگی است که از نظر فیزیولوژیکی، فیزیکی، و روانی تحولات بسیار عمیقی در فرد ایجاد می شود و باعث بهم خوردن نظم جسمانی و روانی نوجوان می گردد. از نظر بهداشت روانی، شناخت این تحولات به‌خصوص در آن قسمت‌ها که تغییرات چشمگیر در رفتار و کردار نوجوانان آشکار می‌گردد پراهمیت است. از عوامل مهم در شکل‌گیری رفتارها، افکار، نگرش‌ها، احساسات، آرزوها و به‌طور کلی شخصیت نوجوانان، ارتباط با دوستان و همسالان است. آموزش همسالان به منظور افزایش دانش، تغییر نگرش، باور‌ها و رفتار‌ها در سطح فردی، گروهی یا سطوح اجتماعی به کار گرفته می‌شود. می توان با برنامه های متناسب با نیازهای نوجوانان و با شیوه­های نوین آموزشی مانند رویکرد گروه همسالان از بسیاری از مشکلات دوران بلوغ پیشگیری کرد. در این مطالعه به بررسی تاثیر آموزش با رویکرد گروه همسالان بر  سلامت روان دختران در دوران بلوغ پرداخته شده است. روش بررسی: در این مطالعه نیمه تجربی 100 نفر از دانش آموزان دختر مقطع راهنمایی شهر تهران در دو گروه 50 تایی آزمایش و شاهد مورد بررسی قرار گرفتند. روش نمونه گیری بصورت تصادفی بود. مداخله با استفاده از رویکرد گروه همسالان انجام گرفت. بعد از گذشت یک ماه و نیم میزان آگاهی و عملکرد از طریق پس آزمون سنجیده و با پیش آزمون در دو گروه مقایسه گردید. برای تجزیه و تحلیل داده ها از آزمونهای آماری کای اسکوئر، تی مستقل و تی زوج­  استفاده شد. یافته­ها: میانگین نمرات آگاهی و عملکرد دختران مبتنی بر رویکرد گروه همسالان به ترتیب از  44/3± 33/27 به 72/2±6/63 و  29/4 ± 73/47 به 63/2 ± 53/88 افزایش یافت (001/0 (P< ولی در گروه شاهد تفاوت معنی داری دیده نشد.  نتیجه گیری: با توجه به نتایج مطالعه مشارکت فعال آموزش گیرندگان و افراد درگیر در موضوع باعث افزایش اثر بخشی آموزشها با محتوای سلامت بلوغ افراد می­شود. بنابراین طراحی برنامه­های آموزشی منظم با شیوه­های مشارکتی و مبتنی بر جامعه هدف مانند  رویکرد گروه همسالان به خصوص در مدارس جهت ارتقای سلامت روانی لازم است.

Title: تبیین تجارب پرستاران از تعارض کار- خانواده و ایفای حقوق کودکان: یک مطالعه کیفی

http://tbj.ssu.ac.ir/article-1-1804-fa.html

سابقه و اهداف: تعارض کار-خانواده از فشارهای ناسازگارانه نقش های خانوادگی و شغلی بوجود می آید ودر بین مشاغل مختلف از جمله پرستاری امری طبیعی و اجتناب ناپذیر تلقی می شود. تعارض پیامدهای منفی متعددی برای سازمانها، افرادو خانواده ها بویژه کودکان دارد . هدف مطالعه تبیین تجارب پرستاران از تعارض کار-خانواده وایفای حقوق کودکان می باشد. روش بررسی : این مطالعه کیفی با مشارکت11پرستار با  نمونه گیری هدفمند، مصاحبه عمیق و بدون ساختارو تجزیه و تحلیل به شیوه رویکرد تحلیل محتوای متعارف انجام شد. یافته ها : یافته های تحقیق شامل 2 درونمایه بود یکی از درونمایه های استحصال شده، تدبیر امور به منظور متعادل سازی با زیر طبقات تدبیر امور خانواده و تدبیر امور کاربود که اشاره به تلاش ها و تدابیر اتخاذ شده توسط پرستاران برای رعایت حقوق کودکان خود داشت .درونمایه دیگر نیازهای غفلت شده کودکان  با زیر طبقات بعد عاطفی، بعد جسمانی و امور تحصیلی بود که اشاره به ناتوانی در مدیریت تعارض و برقراری تعادل بین وظایف خانوادگی و کاری و پیامدهای منفی ناشی از آن بر کودکان در شرایط خاص داشت. نتیجه گیری: پرستاران تلاش هایی را برای به حداقل رساندن تبعات ناشی از تعارض در محیط های خانوادگی از جمله ایفای حقوق کودکان خود انجام می دهند. لیکن در مواردی به دلیل ضعف درمهارتهای فردی مدیریت تعارض و بعضا به دلیل وجود برخی موانع و محدودیت های کاری، پیامد های منفی تعارض آشکار شده و مواردی مثل غفلت از حقوق کودکان  بوقوع می پیوندد که در این رابطه ضرورت ارتقاء مهارت های فردی مدیریت تعارض کار/خانواده و همچنین ارتقاء توانمندی پرسنل در زمینه تدبیر امور خانواده و شناخت نیازها و حقوق اساسی کودکان، در قالب طرح های آموزش بهداشت خانواده از یک طرف، واز طرف دیگر تلاش برای بهبود و اصلاح قوانین کاری و انعطاف پذیری آنان بویژه برای زنان شاغل  برای پیشگیری و به حداقل رساندن عوارض و پیامد های منفی تعارض ؛ توصیه می گردد. .

Title: بررسی کارایی پودراستخوان ماهی مرکب درحذف رنگ راکتیو قرمز198 از محلول های آبی

http://tbj.ssu.ac.ir/article-1-1805-fa.html

چکیده سابقه و اهداف: ترکیبات رنگی از آلاینده های مهم زیست محیطی محسوب  شده که غالباً سمی، سرطانزا و جهش زا بوده و می توانند باعث آلرژی و مشکلات پوستی  شوند. لذا آلاینده های رنگی قبل از تصفیه به محیط زیست باید تصفیه گردند.  هدف از این مطالعه بررسی کارآیی پودر استخوان ماهی مرکب در حذف رنگ راکتیو قرمز 198 از محلولهای آبی و بررسی تأثیر پارامترهای مختلف بر فرآیند جذب آن بوده است. روش بررسی: این مطالعه به صورت تجربی و در مقیاس آزمایشگاهی انجام شده است. در این تحقیق اثر غلظت اولیه رنگ (25 و 50 میلی گرم در لیتر)، جرم جاذب (1، 5/1، 2، 5/2 گرم در 100 میلی لیتر)، زمان واکنش (10، 30، 60، 120، 180، 240 دقیقه) و pH (4، 7، 10)  بر حذف رنگ راکتیو قرمز 198 مورد بررسی قرار گرفت. طول موج حداکثر جذب رنگ (&lambdamax) با استفاده از اسپکتروفتومتر UV/Vis(مدلSP-3000 Plus) تعیین و غلظت مقادیر مجهول رنگ به روش اسپکتروفتومتری تعیین گردید. جاذب مورد نظر در شرایط آزمایشگاهی تهیه و با استفاده از الکهای استاندارد ASTM با اندازه های مش بین 60 و 100 دانه بندی شد. یافته ها: براساس آنالیز طیف جذبی رنگ مورد نظر، طول موج حداکثر جذب(&lambdamax)رنگ راکتیو قرمز 198 درحد 518 نانومتر تعیین گردید. نتایج نشان داد که افزایش دوز جاذب از 2 گرم به 5/2 گرم در 100 میلی لیتر از محلول رنگ با غلظت 25 و50 میلی گرم در لیتر به ترتیب منجر به افزایش راندمان جذب از 85%  به 86 % و از 73 %  به 77%  گردید. افزایش pH اولیه محلول از 4 به 10 به دلیل آزاد شدن ترکیبات کربناته از جاذب به محیط واکنش و تغییر  pH نهایی  محلول به حدود 9 تا 5/9، تأثیر چندانی  بر روی راندمان جذب نداشته و برای غلظت های 25 و 50 میلی گرم در لیتر به ترتیب راندمان جذب از 85%  به 73%  رسیده است. بررسی اثر غلظت اولیه رنگ در حذف نشان داد که با افزایش غلظت اولیه رنگ از 25 به 50 میلی گرم در لیتر با ثابت نگهداشتن دوز جاذب، راندمان جذب از 87%  به 75% کاهش می یابد ولی ظرفیت جذب(qe)از 08/1 به 82/1 افزایش می یابد. به علاوه بیشترین میزان جذب رنگ در 30 دقیقه اولیه واکنش اتفاق افتاد. نتیجه گیری: پودراستخوان ماهی مرکب جاذب طبیعی و ارزان قیمتی است که می توان از آن برای حذف آلاینده های زیست محیطی استفاده کرد.

Title: بررسی میزان آگاهی و نگرش دانشجویان دانشگاه‌های شهرستان یزد در رابطه با بهداشت باروری در سال 1389

http://tbj.ssu.ac.ir/article-1-1806-fa.html

سابقه و اهدف: افراد جوان با خطرهای گوناگونی حاصل از عدم رعایت بهداشت باروری مانند عفونت‌های منتقله جنسی، عفونت HIV، حاملگی زودرس، بیماری و مرگ برای مادران و نوزاد و حاملگی ناخواسته روبرو هستندکه اغلب منجر به سقط غیر ایمن و عوارض ناشی از آن می‌شود. بر اساس آمار تخمینی سازمان بهداشت جهانی حدود 38 درصد از بارداری‌ها در کل جهان نا خواسته بوده است. با توجه به موارد فوق و افزایش موارد حاملگی ناخواسته تا میزان 24 درصد در کشور، ارتقاء رفتارهای مرتبط با بهداشت باروری در جوانان ضروری می‌باشد. هدف از انجام این مطالعه تعیین میزان آگاهی و نگرش دانشجویان دانشگاه‌های شهرستان یزد در رابطه با بهداشت باروری بود. روش بررسی: این مطالعه توصیفی- مقطعی و جامعه مورد پژوهش دانشجویان دانشگاه‌های (یزد، آزاد و علوم پزشکی شهید صدوقی) شهرستان یزد بودند که در یک نمونه 755 نفری به صورت تصادفی طبقه‌ای وارد مطالعه شدند. ابزار گردآوری اطلاعات ﭘﺮﺳﺸﻨﺎﻣﻪ 46 سوالی محقق ساخته با روایی و پایایی معتبر در سه بخش: دموگرافیک 10 سوال، آگاهی 19 سوال و نگرش با 16 گویه و یک سوال در مورد منبع کسب اطلاع بود. داده‌ها توسط نرم افزار آماری SPSS ویرایش هجدهم با کمک آمار توصیفی و آزمون‌های تحلیلی من ویتنی، کروس کال والیس، کای دو و ضریب همبستگی اسپیرمن در سطح معناداری 05/0 مورد آزمون قرار گرفتند. یافته‌ها: از مجموع شرکت کنندگان در این مطالعه 6/48% مرد و 1/82% مجرد و 14% متاهل بودند. 8/38% در دانشگاه آزاد، 4/47% در دانشگاه یزد و 8/13% در دانشگاه علوم پزشکی مشغول به تحصیل بودند. میانگین نمره آگاهی 29/5±45/16 (محدوده نمره 27-0) و نگرش 29/18±17/52 (محدوده نمره80-16) بود. میان میانگین نمرات آگاهی و نگرش دانشجویان با وضعیت تأهل (039/0P=) و (012/0P=)، جنس (009/0P=)و (001/0P=)، مقطع تحصیلی آن‌ها (003/0P=)و (0001/0P=) و نوع دانشگاه محل تحصیل (0001/0P=) و (0001/0P=) اختلاف معناداری وجود داشت. نتیجه گیری: بیش از نیمی از دانشجویان نسبت به بهداشت باروری آشنایی کلی داشتند و به نظر می‌رسد با تاکید بیشتر بر جنبه‌های آموزشی چون آموزش همسان محور در دانشگاه‌ها به توان به نتایج قابل قبول‌تری در زمینه ارتقاء آگاهی و نگرش دانشجویان جهت عملکرد شایسته بهداشتی دست یافت.
